# Hypothyroidism in Noninterferon Treated-HCV Infected Individuals Is Associated with Abnormalities in the Regulation of Th17 Cells

**DOI:** 10.1155/2010/971095

**Published:** 2010-03-08

**Authors:** Luis A. Salazar, Xóchitl Garcia-Samper, Rafael Suarez-Carpio, María C. Jimenez-Martínez, Erika P. Rendón-Huerta, Felipe A. Masso, Teresa I. Fortoul, Luis F. Montaño

**Affiliations:** ^1^Laboratorio de Inmunobiología, Departmento de Biología Celular y Tisular, Facultad de Medicina, Universidad Nacional Autónoma (UNAM), Ciudad Universitaria, CP 04510, DF, Mexico; ^2^Servicio de Gastroenterología, Hospital Regional Lic. Adolfo López Mateos, ISSSTE, Avenida Universidad 2050, Viveros Coyoacan, CP 04526, DF, Mexico; ^3^Unidad de Investigación, Instituto de Oftalmología “Conde de Valenciana”, Chimalpopoca 14, CP 06800, DF, Mexico; ^4^Departamento Bioquímica, Facultad de Medicina, Universidad Nacional Autónoma de México (UNAM), Ciudad Universitaria, CP 04510, DF, Mexico; ^5^Departamento Biologìa Celular, Instituto Nacional de Cardiología “Ignacio Chávez”, Juan Badiano 1, Tlalpan, CP 14080, DF, Mexico

## Abstract

HCV-Ag-specific TH17 cells secrete IL17, a cytokine involved in autoimmune diseases and regulated by IL10 and TGF-b. 5–12% of patients with chronic HCV infection have hypothyroidism. We evaluated the role of these cytokines in this patients by determining serum concentration of TsH, T3, free T4, IL2, IL10, IL12, IL17, TGF-b, anti-TG, TPO, CCP, GBM, and cardiolipin antibodies in 87 chronically noninterferon treated HCV-infected patients. 20 patients (group A) had elevated TsH values (>5 *μ*UI/ml) whereas the remaining 67 (group B) had normal values. The percentage of anti-TPO, TG, GBM, and cardiolipin antibodies in group A patients (33%, 41%, 5% and 5%, resp.) as well as IL17, IL2 and TGF-b concentrations (25 ± 23 pg/ml, 643 ± 572 pg/ml, and 618 ± 221 pg/ml, resp.) were significantly higher than group B. Abnormal Th17 regulation mediated by IL-2 and low TGF-b concentrations is associated with hypothyroidism in chronically-infected HCV patients.

## 1. Introduction

There is ample evidence showing that 7–11% of HCV-infected children and adult individuals have thyroid dysfunction, mainly subclinical hypothyroidism, prior to the initiation of treatment [1, 2]. The association between autoimmune thyroiditis in HCV-infected patients, in particular in subjects with HCV-related mixed cryoglobulinemia, has also been confirmed [3]. Once combined pegIFNalpha2b + ribavirin therapy is initiated, the percentage of patients with thyroid dysfunction goes up to 15–20% [4]. Immunological mechanisms at the origin of this thyroid dysfunction are presumed but the precise mechanism still is unknown. 

IL-17A, a proinflammatory cytokine, is secreted by the CD4+Th17 cells [5]. It is involved in some organ-specific autoimmune diseases such as rheumatoid arthritis, asthma, inflammatory bowel disease, multiple sclerosis, and even organ allograft rejection [6–9], among many others. The main function of IL-17-secreting T cells is to mediate inflammation, by stimulating production of inflammatory cytokines, such as TNF-a, IL-1b, IL-6, and inflammatory chemokines that promote the recruitment of neutrophils and macrophages [10]. Initial evidence suggested that human Th17 cells were regulated by CD4+25+FoxP3+ regulatory T cells, but recent evidence suggests an alternative regulatory mechanism mediated by IL-10 and TGF-b [11, 12]. Ag-specific Th17 cells are induced in HCV-infected patients, but the HCV-NS4 protein also induces the secretion of IL-10 and TGF-b by monocytes [13]. Neutralization of both anti-inflammatory cytokines significantly enhances NS4-specific IL-17 and IFN-gamma production by T cells from HCV-infected donors [14]. Although this may represent a novel immune subversion mechanism by the virus to evade host protective immune responses, it could also be responsible for the thyroid dysfunction. The precise mechanisms of such dysfunction remain obscure. 

An initial evaluation of the thyroid function in chronically infected HCV patients referred to the Gastroenterology Service at Mexico's H.R. “Lic. Adolfo Lopez Mateos”, ISSSTE, showed that a high percentage of patients had subclinical hypothyroidism prior to the initiation of combined treatment. Therefore, our aim was to evaluate the serological status of IL-10, TGF-b and IL-17 in chronically non-interferon treated HCV-infected patients with or without subclinical hypothyroidism.

## 2. Material and Methods

### 2.1. Patients

Eighty seven patients attending an HCV out-patient clinic at HR “Lic. Adolfo Lopez Mateos”, ISSSTE, Mexico, were recruited for this study. Written informed consent was obtained from each patient. The study was approved by the local Research and Ethics Committee. All patients tested positive for antibodies to HCV using an enzyme immunoassay (Abbott Diagnostics). They were all infected as determined by consistently positive HCV RNA serum detection results using a qualitative RT-PCR (Amplicor; Roche Diagnostic Systems). None of the patients (25 males and 62 females) had ever received treatment with pegylated interferon and/or ribavirin. Their mean age was 50.1 years old (31–69). A single experienced histopathologist scored liver biopsy specimens from all the patients. All the patients were catalogued as a class A Child-Pugh score and were selected to initiate pegylated interferon treatment. The control group was conformed by 40 individuals (20 males, 20 females; with a mean age of 48 years old) with no history of liver disease, negative to HCV antibodies and normal liver function tests.

### 2.2. Biochemical Parameters

Blood samples, freshly obtained from each patient one week before treatment was initiated, stood for 5 minutes at room temperature before being centrifuged for 10 minutes at 1800 rpm at 4°C. Plasma and sera was obtained from each patient and aliquots of 0.1 ml were kept at −70°C until use. Plasma levels of IL-2, IL-10, IL-12, IL-17, and TGF-b were determined using commercially available kits purchased from Genzyme and R & D systems (Quantikine) carefully following the manufacturer instructions. The *R* value for the linear regression of the standard curve had to be 0.95 or higher, a lower value was considered inadequate and the data obtained of such plates was eliminated. In order to activate latent TGF-b to immunoreactive TGF-b platelet-poor plasma samples was incubated for 10 min at room temperature with 1 N HCl and then the sample was neutralized with a solution made of 1.2 N NaOH/0.5 M HEPES for another 10 minutes before the determination was performed. Commercially available kits, also from R & D systems were used to determine antithyroglobulin (TG), antithyroid peroxidase (TPO), anticardiolipin, antiglomerular basement membrane (GBM) and anticyclic citrullinated peptide (CCP) antibodies. The plates were read in a Multiskan Ascent (Thermolab systems, Maltham, MA, USA) microplate reader using a 450 and 490 nm filters. Results represent the mean of duplicates. 

Hormone determination (TsH, T3, and free T4) and liver function tests (ALT, AST, Albumin, Immunoglobulin) were performed in the laboratory of the hospital. Subclinical hypothyroidism was defined as a TsH value of ≥4 pg/ml with normal T3 and T4 values. 

The results of all these evaluations in the control group were within the established normal values determined in our laboratory (0.25–4.0 *μ*UI/ml for TsH; 0.6–1.9 ng/ml for T3; 0.7–1.8 ng/dl for free T4; 2–40 UI/ml for AST and 2–40 UI/ml for ALT). The results obtained in the control samples for the different cytokines were 6.8 ± 2.7 pg/ml for IL-2; 4.9 ± 1.4 pg/ml for IL-10; 52.7 ± 8.1 pg/ml for IL-12; 6.75 ± 1.14 pg/ml for IL-17, and 398 ± 271 pg/ml for TGF-b1). The normal value for anti-TG is 0–34 IU/ml, 0-12 IU/ml for anti-TPO, >10 IU/ml for anti-cardiolipin, and 0–7 IU/ml for anti-GBM and anti-CCP antibodies.

### 2.3. Statistical Analysis

Data is expressed as mean ± standard deviation of the mean. Differences between groups were evaluated by Student's *t* test, confidence interval at the 95% value, Tukey test and Kruskal-Wallis one way analysis of variance on Ranks, and Holm Sidak method. Data was analyzed with the SPSS 12.0 release. A *P* < .05 was considered statistically significant.

## 3. Results

The mean age of male patients was 45.7 years old whereas that of the females was 51.8 years old. The predominant HCV genotype was 1b (42 patients) followed by 1a (18 patients), 2b (17 patients), 2a (9 patients), and one patient with genotype 4.  Table 1 shows the clinic-serological parameters of all the patients. Seventy percent of the patients with elevated TsH (6.45 ± 2.21 *μ*IU/ml) (group A) were females; the percentage of female patients in the group with normal TsH values (2.58 ± 0.95 *μ*UI/ml) (group B) was similar (71%). The difference in TsH values between both groups was highly significant (*P* < .0001) as well as between group A and control group (*P* < .001; according to the all pairwise multiple comparison procedures-Holm-Sidak method). There were no statistical differences in TsH values related to gender (5.82 ± 1.73 *μ*UI/ml for males and 6.71 ± 2.40 *μ*UI/ml for females in group A versus 2.26 ± 0.76 *μ*UI/ml for males and 2.68 ± 0.99 *μ*UI/ml for females, in group B). Similarly there were no statistical significant differences between both groups in relation to viral load. Although the values of the following markers were statistically different from those of the control group, there were no statistical significant differences between group A and group B results in relation to AST, ALT, T3, and free T4. 

Eight group A patients had elevated anti-TG antibodies (41%). Six of those also had elevated anti-TPO antibodies (33%). One patient with normal anti-TG, anti-TPO, and anti-CCP values had elevated anti-GBM and anti-cardiolipin antibodies. This patient had consistently low platelet counts but had no history of miscarriage or deep vein thrombosis. None of the anti-TPO nor anti-TG positive patients in group A had signs or symptoms of Hashimoto's thyroiditis. The percentage of autoantibodies in group B was 33% for anti-TG antibodies, and 7% for anti-TPO. None of the patients in group B had elevated values of anti-GBM or anti-CCP, and only one had elevated anti-cardiolipin antibodies. Interestingly, the latter patient was also positive for anti-TPO antibodies and had deep vein thrombosis of the lower extremities. 

In relation to the cytokines it was interesting to observe that although the concentrations in HCV+ patients were significantly higher than those found in the control group for all the cytokines, the intergroup differences (group A individuals versus group B) for IL-10 (14.83 ± 2.98 pg/ml versus 15.27 ± 1.70 pg/ml) and IL-12 (271.59 ± 196.43 pg/ml versus 221.13 ± 101.60 pg/ml) were not statistically significant. The cytokines that showed a highly statistically significant difference between group A and group B patients were: IL-2, IL-17, and TGF-b (Table 1). The Kruskal-Wallis analysis of variance at the 0.05 level of significance for IL-2 showed a median value of 611 pg/mL for group A, 195 pg/mL for group B, and 7.36 pg/mL for the control group (*P* < .001); the significance of these differences between groups A and B with the control was confirmed by the Tukey test (*P* < .05). As far as IL-17 was concerned, the Kruskal-Wallis analysis of variance showed a median value of 19.4 pg/mL for group A, 18.5 pg/mL for group B, and 8.9 pg/mL for the control group (*P* < .001); similarly to IL-2, IL-17 results were also confirmed by the Tukey test. There were no differences in IL-17 concentration in both groups in relation to the viral genotype. Finally, TGF-b values passed the normality test and the one way analysis of variance demonstrated a significant difference between groups A and B with the control group (*P* < .001) but not between group A and group B. It was interesting to observe that TGF-b concentration in group A patients was 38% below that of group B patients.

## 4. Discussion

HCV infection has been related to various autoimmune disorders including thyroid dysfunction [1–4] and papillary thyroid cancer [15]. Patients with HCV-related mixed cryoglobulinemia also show thyroid dysfunction [3]. In summary, the presence of hypothyroidism in chronically HCV-infected individuals, either under treatment or not, has been well documented [1, 16, 17]. One interesting observation was the high percentage of HCV-infected patients having hypothyroidism (23%) versus an average 12% cited in the literature; this could be secondary to the significantly higher frequency of thyroid microsomal antibodies in Mexican women in comparison with Caucasian or black women [18] thus representing a higher predisposition to autoimmunity against thyroid gland. We found autoimmune thyroid involvement as judged from serum auto-antibodies and Tsh values in 20 of the 87 patients that we analyzed. The percentage of individuals in the subclinical hypothyroidism group with autoantibodies was significantly higher than the percentage found in normothyroid HCV+ patients and control. None of our patients had manifestations of Hashimoto's disease and only one of the female patients in the hypothyroid group, had a pattern compatible with an antiphospholipids syndrome, an association found in 20% of HCV-infected patients [19]. The percentage of females with autoimmune disease in our study (70%) is identical to that found in a multicenter international study [20, 21]. Nevertheless, it has recently been suggested that the presence of autoantibodies in HCV-infected patients do not appear to be of clinical importance [22]. One patient in each group had elevated anticardiolipin antibodies but none of them suffered stroke, although the patient with deep vein thrombosis is a strong candidate. This association has already been described [23]. 

The thyroid dysfunction in HCV-infected patients has been associated to interleukin 2 [24–26], a cytokine secreted primarily by Th1 cells. Patients with non-HCV-related diseases being treated with high doses of IL-2 also develop thyroid dysfunction [27, 28]. The increased interleukin 2 serum concentration determined in our non-IL-2 or IFN-treated HCV+ hypothyroid patients, corroborates the above mentioned findings. Interleukin 2 is known to expand Th17 cells in some autoimmune diseases [29].

A key cytokine for IL-2 production and Th1 cell differentiation is IL-12 [30, 31]. Although the core protein of HCV seems to have a suppressive action on IL-12 production at the transcriptional level [32], our results showed that the concentration of IL-12 was similar in both of our HCV-infected groups, but considerably higher than the control group. This suggests that hypothyroidism was not related to interleukin-2 despite the fact that transgenic mice with IL-12 serum concentrations in the 150 pg/ml range develop a moderate primary hypothyroidism [33]. IL-12 is responsible for inducing a Th17 response [5, 34].

The Th17 response is characterized by a serum increase in interleukin-17A, commonly known as IL-17. This cytokine, secreted by the CD4+ T cells known as Th17, is also secreted by some CD8+ T cells, NKT, alpha-beta or gamma-delta T cells, eosinophils, neutrophils, and monocytes [35–37]. Th17 cells are mainly linked to autoimmune diseases, namely, multiple sclerosis, inflammatory bowel disease, rheumatoid arthritis, Lyme disease, psoriasis, and uveitis [29, 38]. Of the five different IL-17 receptors (A to E), IL-17 only bind to IL-17RA and C [35, 39]. The pituitary gland and the thyroid, in contrast to hepatocytes, express IL-17RC [40]. The regulation of Th17 cells is basically mediated by CD4+CD25+FoxP3+ T reg cells which are capable of inducing anergy towards self- and alloantigens, thus playing an important role in autoimmunity [41–43]. Nevertheless, a new regulatory mechanism mediated by IL-10 and TGF-b, both of which suppress IL-17 production [11, 12], has been suggested [44]. Our hypothyroid patients had similar amounts of IL-10 than the normo thyroid group but lower TGF-b serum concentration. Therefore, the only abnormality that could explain the high IL-17 serum concentration and the thyroid dysfunction was related to alterations in the FoxP3+ Treg cells, probably through enhanced Stat3 phosphorylation [36, 45]. Nevertheless, recent evidence shows that TGF-beta orchestrates Th17 cell differentiation in a concentration-dependent manner. Low TGF-b concentrations favors Th17 cell differentiation whereas high TGF-b concentrations favors Foxp3+ Treg cell differentiation [43]. The former is accompanied by high levels of IL-17, as we observed in our hypothyroid patients, whereas the latter is accompanied by a tighter regulation of IL-17 secretion, as we observed in our normothyroid patients. Despite TGF-b apparent importance, Th17 differentiation depends basically on the presence of IL-1 and IL-6 [46]. It has recently been shown that the proportion of peripheral Th17 cells in patients with autoimmune thyroid disease is higher than in control subjects [47].

Although there is evidence showing that IL-2 can inhibit human Th17 cell differentiation through a STAT-5 mediated pathway and enhanced TGF-b-induced FoxP3 expression [48, 49], a recent report by Deknuydt et al. [50] show that CD4+CD25+ FOXP3+ T regulatory cells in the presence of IL-2, in an inflammatory microenvironment, can be converted into proinflammatory Th17 cells. This could explain the link between inflammation, high IL-2 and IL-17 concentrations, and autoimmunity in our patients. Interleukin 17 is also involved in human alcoholic liver disease, an entity that shares some features with autoimmune diseases [51]. 

The viral load in our patients could be definitively considered low and such viral loads have been linked with the development of auto antibodies due to the lowering of the B cell activation threshold or by inducing self-reactivity through a mechanism of molecular mimicry [52, 53].

## 5. Conclusions

We believe that our results point towards an abnormality in the T regulatory cells, induced by the hepatitis C virus. The fact that not all the HCV-infected patients develop an full blown autoimmune disease suggests that there are many individual variations that go beyond the viral load, viral genotype, presence of auto antibodies or liver biochemical alterations. We must not forget that the HCV does replicate within the thyroid tissue [54] and that the evolution of the HCV infection is related to distinct immune cell cytokine expression profiles. Recently it has been postulated that a Th1cell mediated immune response underpins the association of chronic HCV infection with endocrine disease [55], our results suggest that a Th17 immune response should also be considered.

## Figures and Tables

**Table 1:** Clinicoserological findings in hypo- and normothyroidism HCV patients.

	Group A (*n* = 20)	Group B (*n* = 67)	Control (*n* = 40)	*P* value
Females/males	14/6	48/19	20/20	—
Age, mean ±SD (years)	49.8 ± 9.7	49.8 ± 12.2	51.2 ± 14.9	NS
HCV genotype				
1	12	45	0	—
2	7	21	0	—
4		1		—
Viral load HCV RNA (<50 IU/mL)	489,731 ± 381,451	399,129 ± 319,863	Negative	NS
ALT (IU/mL)	99.75 ± 65.58	81.23 ± 55.38	25 ± 1.62	NS
AST (IU/mL)	81.37 ± 54.31	72.28 ± 54.33	32 ± 1.33	NS
Platelets (k/*μ*L)	162 ± 73.3	206.6 ± 85.1	309 ± 95	NS
Albumine (g/dL)	4 ± 0.48	3.9 ± 0.8	4.1 ± 0.4	NS
TSH (*μ*IU/mL)				
Total	6.45 ± 2.21	2.58 ± 0.95		
Males	5.82 ± 1.73	2.26 ± 0.76	2.6 ± 1.1	< .0001
Females	6.71 ± 2.40	2.68 ± 0.99		
T3 (ng/mL)	1.4 ± 0.9	1.51± 0.6	1.3 ± 0.5	NS
T4F(ng/dL)	1.18 ± 0.4	1.51 ± 0.6	0.9 ± 0.3	NS
Anti-thyroperoxidase antibody (AbTPO)*	33%	7%	Negative	< .001
Anti-thyroglobulin antibody (AbTg)*	41%	33%	Negative	< .01
Anticyclic Citrullinated Peptides antibody (CCP)	Negative	Negative	Negative	—
Glomerular Basement Membrane (GBM)	Negative	Negative	Negative	—
Cardiolipin	Negative	Negative	Negative	
IL-2 (pg/mL)	643.07 ± 572.66**	292.89 ± 335.91	6.8 ± 2.7	< .0009
IL-10 (pg/mL)	14.83 ± 2.98	15.27 ± 1.70	4.9 ± 1.4	NS
IL-12 (pg/mL)	271.59 ± 2.98	221.13 ± 101.60	52.7 ± 8.1	NS
IL-17 (pg/mL)	25.88 ± 23.14**	14.60 ± 7.83	6.75 ± 1.14	< .001
TGF-*β* (pg/mL)	618.97 ± 221.89**	850.14 ± 415.64	398 ± 271	.019

Group A are the hypothyroid HCV+ patients whereas group B are the normothyroid HCV+ patients.

*Percentage of patients with values above the normal range.

**P  <  .01 between group A and group B.
